# Genome‐scale CRISPR screening identifies cell cycle and protein ubiquitination processes as druggable targets for erlotinib‐resistant lung cancer

**DOI:** 10.1002/1878-0261.12853

**Published:** 2020-11-28

**Authors:** Jieun Lee, Ahyoung Choi, Sung‐Yup Cho, Yukyung Jun, Deukchae Na, Ahra Lee, Giyong Jang, Jee Young Kwon, Jaesang Kim, Sanghyuk Lee, Charles Lee

**Affiliations:** ^1^ Department of Life Science Ewha Womans University Seoul Korea; ^2^ Ewha‐JAX Cancer Immunotherapy Research Center, Ewha Womans University Seoul Korea; ^3^ Department of Bio‐Information Science Ewha Womans University Seoul Korea; ^4^ Department of Biochemistry and Molecular Biology Seoul National University College of Medicine Seoul Korea; ^5^ Department of Biomedical Sciences Seoul National University College of Medicine Korea; ^6^ The Jackson Laboratory for Genomic Medicine Farmington CT USA; ^7^ Ewha Institute of Convergence Medicine, Ewha Womans University Mokdong Hospital Seoul Korea; ^8^ Precision Medicine Center The First Affiliated Hospital of Xi’an Jiaotong University Xi'an China

**Keywords:** cell cycle process, CRISPR/Cas9 screening, erlotinib resistance, lung cancer, protein ubiquitination pathway

## Abstract

Erlotinib is highly effective in lung cancer patients with epidermal growth factor receptor (EGFR) mutations. However, despite initial favorable responses, most patients rapidly develop resistance to erlotinib soon after the initial treatment. This study aims to identify new genes and pathways associated with erlotinib resistance mechanisms in order to develop novel therapeutic strategies. Here, we induced knockout (KO) mutations in erlotinib‐resistant human lung cancer cells (NCI‐H820) using a genome‐scale CRISPR‐Cas9 sgRNA library to screen for genes involved in erlotinib susceptibility. The spectrum of sgRNAs incorporated among erlotinib‐treated cells was substantially different to that of the untreated cells. Gene set analyses showed a significant depletion of ‘cell cycle process’ and ‘protein ubiquitination pathway’ genes among erlotinib‐treated cells. Chemical inhibitors targeting genes in these two pathways, such as nutlin‐3 and carfilzomib, increased cancer cell death when combined with erlotinib in both *in vitro* cell line and *in vivo* patient‐derived xenograft experiments. Therefore, we propose that targeting cell cycle processes or protein ubiquitination pathways are promising treatment strategies for overcoming resistance to EGFR inhibitors in lung cancer.

## Graphical abstract

Here, we discovered that targeting cell cycle processes or protein ubiquitination pathways are promising treatment strategies for overcoming resistance to EGFR inhibitors in lung cancer using a genome‐scale CRISPR‐Cas9 screening. Combination therapies targeting each of these two processes such as nutlin‐3 and carfilzomib increased cancer cell death when combined with erlotinib in both *in vitro* and *in vivo* experiments.

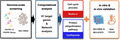

**Abbreviations**
ATCC: American Type Culture Collection
edgeR: bioconductor software package for examining differential expression of replicated count data
EGFR: epidermal growth factor receptor
GeCKO: genome‐scale CRISPR/Cas9 knockout
HGF: hepatocyte growth factor
MAGeCK‐VISPR algorithm: comprehensive quality control analysis and visualization pipeline for CRISPR/Cas9 screens based on MAGeCK VISPR
MOI: multiplicity of infection
NSCLC: non‐small‐cell lung cancer
SCLC: small cell lung cancer
sgRNA: single‐guide RNA
TKIs: tyrosine kinase inhibitors

## 1 Introduction

Lung cancer is the most commonly diagnosed cancer in the world and a leading cause of cancer‐related deaths [1]. A number of new targeted therapies have been developed for lung cancers with mutations in specific genes, such as epidermal growth factor receptor (EGFR), which is known to control cell growth and proliferation [2]. Mutations of this receptor can lead to activation of downstream signaling cascades such as cell proliferation, apoptosis, and migration, thus contributing to tumorigenesis and metastasis [3]. Many tyrosine kinase inhibitors (TKIs) have been developed to suppress the tumor‐promoting properties caused by EGFR mutations in non‐small‐cell lung cancer (NSCLC) patients [4, 5]. Among the EGFR‐targeting TKIs, erlotinib is widely used for both localized and metastatic NSCLC patients [6, 7] because it has relatively few side effects and high efficacy [8]. However, many patients subsequently develop resistance to erlotinib treatment.

The mechanism of acquired resistance to first‐generation EGFR‐TKIs (such as erlotinib) is the occurrence of a secondary EGFR kinase domain mutation, such as the T790M substitution in exon 20—which accounts for about half of the erlotinib‐resistant cases [9]. Other genomic mutations in tumor cells that may contribute to EGFR‐TKIs resistance include amplification of the MET oncogene [10], overexpression of hepatocyte growth factor (HGF), amplification of the ERBB2 gene [11], aberrant downstream pathways (e.g., AKT mutations and PTEN loss), impairment of the EGFR‐TKIs‐mediated apoptosis pathway (e.g., BCL2L11/BIM deletion polymorphism), and histological transformation to small cell lung cancer (SCLC) [9]. Increasing the survival benefits from first‐line treatments in NSCLC patients with EGFR mutations and delaying the occurrence of resistance are two critical tasks that could be solved by EGFR‐TKI‐based combination therapies, including combinations with various chemo‐agents, targeted cancer drugs, and even immunotherapeutic methods.

Genetic screening using CRISPR‐Cas9 can be used for rapidly identifying driver genes associated with various hallmarks of cancer progression [12]. The CRISPR‐Cas9 system is based on RNA‐guided nucleases where a single‐guide RNA (sgRNA) directs the Cas9 nuclease to cause double‐stranded cleavage of matching target DNA sequences [13]. The ease of retargeting Cas9 by simply designing short guide RNA sequences to every human gene enables large‐scale unbiased genome perturbation experiments that probe gene function or identify causal genetic variants [14]. The genome‐wide screen of loss of function previously used an RNAi‐based approach, but this approach only causes partial knockdown, has extensive off‐target effects, and is limited to transcribed genes [15]. By contrast, Cas9‐mediated pooled sgRNA screens have provided enhanced screening sensitivity as well as consistency and can be designed to target nearly any DNA sequence [16, 17].

Here, we performed a CRISPR‐Cas9 loss‐of‐function screening experiment followed by sgRNA sequencing to identify genes whose knockout restores erlotinib sensitivity in an erlotinib‐resistant lung cancer cell line. Our sgRNA screen shows cell cycle processes and ubiquitin regulation pathways as specific biological processes that can sensitize erlotinib‐resistant cells. Furthermore, our *in vitro* and *in vivo* data showed a synergistic anticancer effect of chemical inhibitors for specific genes in these two pathways, when used in combination with erlotinib. These findings suggest that combination therapies that include chemical inhibitors to cell cycle processes and ubiquitin regulation pathways can serve as novel treatment strategies to overcome erlotinib resistance for EGFR mutant patients with NSCLC.

## 2 Materials and methods

### 2.1 Cell culture and reagents

Human lung cancer cell lines (NCI‐H820, NCI‐H1975) were purchased from the American Type Culture Collection (ATCC). All cell lines were cultured as previously described [18], and mycoplasma testing was routinely performed using e‐Myco™ plus Mycoplasma PCR Detection Kit (Intron, Korea), verifying that the cells were mycoplasma free. Anti‐PARP, anticleaved caspase 3, anti‐AKT, anti‐phospho‐AKT, anti‐ERK, and anti‐phospho‐ERK antibodies were purchased from Cell Signaling Technology. Erlotinib, SJ‐172550, nutlin‐3, and carfilzomib were obtained from APExBIO. Bortezomib, quinacrine, brefeldin A, and ouabain were purchased from Selleckchem (Houston, TX, USA), and N‐ethylmaleimide was obtained from the Sigma‐Aldrich Corporation (Sigma, St. Louis, MO, USA).

### 2.2 Lentiviral library amplification and cell transduction

The human GeCKO lentiviral pooled library, lentiCRISPR v2, was purchased from Addgene (Watertown, MA, USA) (cat # 1000000048) as two half‐libraries (A and B). The library was amplified according to the manufacturer’s recommendations [17]. Briefly, the library plasmid DNA were transformed using electroporation in Endura electrocompetent cells (Lucigen, Middleton, WI, USA). Colonies were recovered from the plates, and plasmid DNA was extracted using the Endotoxin‐Free Plasmid Maxiprep kit (Qiagen, Germantown, MD, USA).

To produce lentivirus, twelve T‐75 flasks of 293FT cells (Invitrogen, Carlsbad, CA, USA) were seeded at ~ 70 % confluence the day before transfection in D10 media (DMEM supplemented with 10 % fetal bovine serum). During the transfection, media was removed and 4 mL of prewarmed reduced serum OptiMEM media (Gibco, Waltham, MA, USA) was added to each flask. Transfection was performed using Lipofectamine 2000 (Invitrogen, Carlsbad, CA, USA). For each flask, plasmid DNA were cotransfected with 6 μg of lentiCRISPRv2 half‐library A or B with 3 μg of pCMV‐VSVg and 4.5 μg of psPAX2 (Addgene) in 293FT cell. The complete mixture was incubated for 15 min before being added to cells. After 6 h, the media was changed to 12 mL D10 supplemented. After 48 h, supernatants from the transfected 293FT cells were harvested and centrifuged at 3000 rpm at 4 °C for 10 min to remove pellet cell debris. The supernatant was concentrated using Lenti‐X concentrator (Clontech, Mountain View, CA, USA) according to the manufacturer's protocol. Pooled lentiviral libraries are transduced to 1 × 10^8^ NCI‐H820 cells with 3 × 10^6^ cells plated per transduction well. The multiplicity of infection (MOI) is about 0.3–0.4 to insure that most cells receive only one stably integrated RNA guide. To select the cells containing sgRNA, 1 μg·mL^−1^ puromycin was added to cells for 3 days. The GeCKO v2 libraries target 19,050 human coding genes with 6 sgRNAs per gene [19], and each group contained at least 1.2 × 10^7^ cells (coverage 100x).

### 2.3 Negative screening of erlotinib‐resistant cells

Freshly prepared NCI‐H820 cells (~2 × 10^7^ cells) containing GeCKO libraries were split into three plates with each portion containing a minimum of 3 × 10^6^ cells. The first portion were frozen down for genomic DNA analysis to profile the sgRNA diversity after transfection (D0), the second portion cultured in DMSO without erlotinib for 7 (D7) or 14 days (D14) as control samples, and the third portion cultured with 1 μm erlotinib for 7 (E7) or 14 days (E14). Erlotinib‐treated cells and control cells were passaged in fresh media with and without 1 μm erlotinib, respectively. At this time, the IC_50_ of NCI‐H820 cells for erlotinib was more than 100 μm. The screening experiment was performed in duplicate. All samples were subjected to sgRNA sequencing as follows.

### 2.4 sgRNA sequencing and data analysis

Frozen cell pellets were thawed, and genomic DNA was extracted with a Blood & Cell Culture Midi kit (Qiagen). The amplification of sgRNA target sequences for deep sequencing was performed as described [17]. All PCR products obtained from each sample were run on an agarose gel, gel‐extracted, and purified according to the manufacturer’s recommendations.

The resulting libraries were sequenced with single‐end 100‐bp reads on a HiSeq2500 (Illumina, San Diego, CA, USA). Raw sequencing data were preprocessed using the fastx toolkit (http://hannonlab.cshl.edu/fastx_toolkit/) which included the fastq_quality_filter to remove low‐quality reads. Resulting reads were trimmed to remove the constant portion of the sgRNA sequences with CRISPR.sgRNA_read_trimmer in the GenePattern Module Archive (http://www.gparc.org/) and then aligned to the GeCKO v2 sgRNA library using bowtie 1 (v1.1.1) under default settings. After alignment, the number of uniquely aligned reads for each library sequence was calculated by CRISPR.single_sgRNA_count in GParc. The raw read counts were normalized as follows: normalized reads per sgRNA = (reads per sgRNA/total reads for all sgRNAs in sample) × 10^6^ + 1.

In our negative selection screening, we looked for sgRNAs whose number gets depleted in 14 days of erlotinib treatment. Thus, we filtered out sgRNAs that had zero read count in 14 days of DMSO treatment (i.e. without erlotinib treatment). Then, we calculated the ‘beta score’ that represents the extent of selection in a manner similar to the term of ‘log fold‐change’ using MAGeCK‐VISPR (https://bitbucket.org/liulab/mageck‐vispr) program. The MAGeCK‐VISPR algorithm compares the sgRNA abundance of all sgRNAs targeting a gene across different conditions and assigns each gene a beta score of essentialities in each condition compared with the controls. We used the cells at day 0 (D0) as the control samples in calculating the beta score. Genes with beta scores among the highest and lowest 2% were selected as differential between control and treatment samples, and these genes were further subjected to k‐means clustering. Alternatively, we also obtained the differentially expressed sgRNAs between cells at 14 days of DMSO (D14) or erlotinib treatment (E14) using edgeR (version 3.4.2) with the normalized read counts. We applied both the differential expression (FDR < 0.05 and log_2_FoldChange < −1) and the abundance (number of sgRNAs per gene>= 2) criteria to screen for differential sgRNAs.

### 2.5 Functional enrichment and survival analyses

Cytoscape StringApp version 1.5.0 (http://apps.cytoscape.org/apps/stringapp) was used for network expansion [20] and functional enrichment analysis. Starting with the 81 top candidate genes obtained from sgRNA screening, we obtained the expanded network using a confidence score of 0.4 (medium confidence) and the maximum number of interactions to 5 best scoring hits. Then, we used the functional enrichment analysis module in STRING to obtain over‐represented terms of the expanded network nodes in the gene ontology (GO) terms for biological processes. In addition, we also computed the GO enriched terms for 81 top candidate genes using the MSigDB (version 6.2) analysis tool (http://software.broadinstitute.org/gsea/index.jsp) with an FDR cutoff of < 0.05 [21]. As for the survival analysis, we used the OncoLnc website (http://www.oncolnc.org/) to perform Kaplan–Meier analyses with the *P*‐value of log‐rank test < 0.05 for the TCGA lung adenocarcinoma (LUAD) cohort.

### 2.6 Validation of the five candidate genes using CRISPR/cas9 sgRNA

For erlotinib‐sensitive validation study, we produce lentiCRISPR with sgRNAs targeting five candidate genes and one nontargeting control (sgNTC). The sgRNA sequence referred to the human GeCKO lentiviral pooled library sequence (Table S1). The erlotinib sensitivity was measured by *in vitro* cytotoxicity assay. Cell survival at different erlotinib doses was determined by EzCytox WST assay kit (Daeil Lab, Seoul, Korea) and mRNA quantification using real‐time PCR.

### 2.7 In vitro cytotoxicity assay

For the cell viability assays, approximately 5000 cells were plated in each well of a 96‐well plate and incubated at 37 °C with 5% CO_2_ for 1 day then added with the indicated drugs in triplicate at serially diluted concentrations with 100 μL medium, respectively. Cells were treated with the following reagents at the indicated final concentration: erlotinib (50 mM), SJ‐172550 (100 mM), nutlin‐3 (100 mM) carfilzomib (10 mM), bortezomib (20 mM), quinacrine (100 mM), brefeldin A (1 mM), and ouabain (100 mM) for 72 h and examined for cell viability using the EzCytox WST assay kit. Cell viabilities were estimated as relative values compared to the untreated controls. Drug synergism was quantified by calculating the combination index (CI) based on the multiple drug effect equation. The CI for each concentration of drugs was calculated by compusyn Software (https://www.combosyn.com/) where the CI value < 0.9 was indicative of synergism.

### 2.8 Flow cytometry (FACS) analysis

The NCI‐H820 cells were seeded into 60‐mm culture dishes (1 × 10^6^ cells/well) and were treated with the indicated drugs. The treated cells were collected and washed in phosphate‐buffered saline (PBS). The apoptotic cells were analyzed by FITC Annexin V apoptosis Detection Kit (BD Biosciences, San Jose, CA, USA) according to the manufacturer’s protocol. Stained cells were analyzed with a FACScan flow cytometer (Becton Dickinson, Midland, ON, Canada).

### 2.9 Western blot analysis

Cell lysates were prepared using RIPA buffer (Thermo Scientific) with protease inhibitor cocktail (Roche) and phosphatase inhibitor cocktail (Roche, Basel, Switzerland), incubated for 15 min on ice and centrifuged at 16 800 ***g*** for 10 min at 4 °C. The bicinchoninic acid (BCA) method (Thermo Scientific, Waltham, MA, USA) was used to determine the protein concentration. Proteins were resolved by SDS/PAGE and transferred to polyvinyl difluoride membrane. After blocking with 5% skim milk, membranes were individually probed with anti‐phospho‐AKT (Cell Signaling Technology, Danvers, MA, USA), anti‐phospho‐ERK (Cell Signaling Technology), anticleaved caspase 3 (Cell Signaling Technology), anti‐PARP (Cell Signaling Technology), anti‐p21 (Cell Signaling Technology), anti‐MDM2 (Cell Signaling Technology), anti‐MDM4 (Cell Signaling Technology), anti‐p53 (Cell Signaling Technology), anti‐PUMA (Cell Signaling Technology), and anti‐actin (Sigma‐Aldrich Corporation) antibodies. The membranes were washed and incubated with horseradish peroxidase‐conjugated secondary antibodies, followed by enhanced chemiluminescence detection according to the manufacturer’s instructions (ThermoFisher).

### 2.10 *In vivo* experiments

All animal procedures were approved by The Jackson Laboratory Institutional Animal Care and Use Committee, protocol 17‐0028. The patient‐derived xenograft (PDX) LG1049 lung cancer model containing the EGFR T790M mutation was purchased from The Jackson Laboratory (Bar Harbor, Maine). The surgically resected tissues were minced into pieces approximately ~ 2 mm in size and injected into the flanks of NOD/SCID/IL‐2γ‐receptor null (NSG) female mice. Drug treatments began after tumors reached approximately 150–200 mm^3^. Mice were randomly divided into 8 treatment groups consisting of 5 mice in each group: (1) vehicle only, (2) erlotinib only, (3) nutlin‐3 only, (4) erlotinib plus nutlin‐3, (5) carfilzomib only, (6) erlotinib plus carfilzomib, (7) SJ‐172550, and (8) erlotinib plus SJ‐172550. The following drugs and dosing schedules were used: erlotinib: 25 mg·kg^−1^ diluted in 0.5% methylcellulose solution, oral gavage daily; nutlin‐3 and SJ‐172550: 20 mg·kg^−1^ diluted in 10% DMSO, 9% ethanol, 27% polyethylene glycol (PEG) 400, 54% Phosal 50 PG, intraperitoneal injection once every two days; carfilzomib: 1.5 mg·kg^−1^ diluted in 2% DMSO, 30% PEG300, and 2% Tween‐80, intravenous injection once a week. Mouse weight monitoring and sampling were performed as previously described [22].

### 2.11 Statistical analysis

Statistical analyses were performed using prism 4.0 (GraphPad Software, San Diego, CA, USA). Differences between two variables were assessed by an unpaired Student’s *t*‐test. An observation was considered statistically significant if the *P*‐value was less than 0.05.

## 3 Results

### 3.1 Genome‐scale CRISPR screening for erlotinib‐sensitizing genes identified 81 candidate target genes

**Fig. 1:**
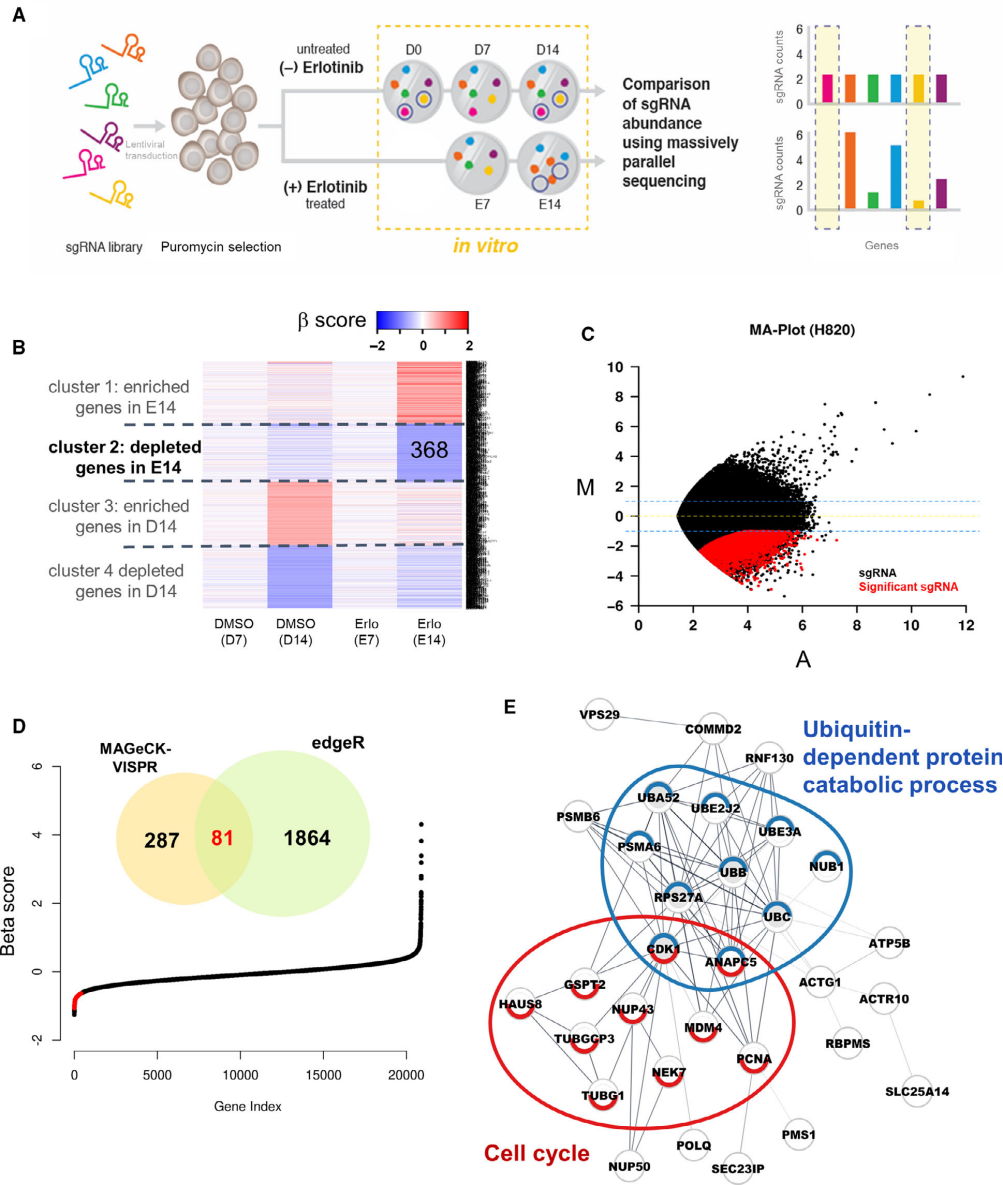
Genome‐scale negative selection screening of erlotinib‐sensitive related genes. (A) Schematic representation of the loss‐of‐function screen using the human genome‐scale CRISPR/Cas9 knockout library. D0, D7, and D14 refer to puromycin‐selected day 0 cells (D0), control day 7 cells (D7) with DMSO, and control day 14 cells (D14) with DMSO, respectively. E7 and E14 refer to erlotinib‐treated day 7 cells (E7) and erlotinib‐treated day 14 cells (E14), respectively. (B) A heatmap of beta scores from MAGeCK‐VISPR on the NCI‐H820 knockout dataset. Using k‐means clustering (k = 4), we identified four clusters. Only genes among the highest or lowest 2% β scores in 14‐day DMSO or erlotinib treatment conditions are shown in the heatmap. (C) The MA plot showing enrichment of specific sgRNAs after erlotinib treatment (*M* = log_2_ E14/D14, *A* = ½log_2_ E14*D14). Red dots (*n* = 4183) indicate sgRNAs significantly depleted after 14 days of erlotinib treatment. (D) Venn diagram showing the overlap of genes depleted between the two methods: MAGeCK‐VISPR and edgeR, in the NCI‐H820 lung cancer cell line. The overlapping genes are indicated with the red dots in the beta score plot of E14 data. (E) STRING network analysis with the 81 commonly depleted genes. Genes involved in the cell cycle process and ubiquitin‐dependent protein catabolic process are indicated in red and blue with colored boundaries, respectively. Gray nodes are the linker genes from the STRING network, which are not differentially expressed.

We applied a genetic screening approach to identify candidate genes whose loss‐of‐function would lead to acquired sensitivity to erlotinib in lung cancer cells that are erlotinib‐resistant. The idea was to use a pool of sgRNA‐expressing lentivirus to generate a library of knockout cells that could be screened for sensitivity to erlotinib via negative selection (Fig. 1A). We transduced an erlotinib‐resistant human lung cancer cell line (NCI‐H820) with the GeCKO v2 library at a multiplicity of infection (MOI) of 0.3. The GeCKO v2 libraries consisted of 123 411 unique sgRNAs for 19 050 genes in the human genome [17]. Cells transduced with lentiviral vectors were maintained with or without erlotinib for 14 days, and residual sgRNA abundance was measured by counting sgRNA numbers using high‐throughput sequencing. After 14 days of erlotinib treatment, the spectrum of sgRNAs incorporated among the erlotinib‐treated cells was significantly different from both erlotinib‐treated cells grown for 7 days, and untreated cells (Figs S1A, S1B**)**.

The GeCKO v2 library contains 6 sgRNAs per gene but the knockout efficiency of each sgRNA was unknown. Therefore, we analyzed the sgRNA screening data using two levels of analyses: (1) gene unit analysis with MAGeCK‐VISPR (Fig. 1B) [23] and (2) sgRNA unit analysis with edgeR (Fig. 1C). MAGeCK‐VISPR estimates the sgRNA abundance (beta score) at the gene level, taking replicates into consideration. Positive (or negative) beta scores mean positive (or negative) selections for the gene at each condition compared with the control samples at day 0 (D0). Genes with beta scores among the highest or lowest 2% were selected as highly variable genes, and these were further subjected to k‐means clustering (*k* = 4) (Fig. 1B; Table S2). We identified 368 genes that showed strong negative selection at 14 days of erlotinib treatment, implying that cells transduced with sgRNAs for these genes became more sensitive to erlotinib treatment. Next, we compared the abundance of individual sgRNAs between cells with (E14) or without erlotinib treatment for 14 days (D14) using the edgeR program. We identified 4183 sgRNAs for 1945 genes whose abundance was significantly reduced in erlotinib‐treated cells (false discovery rate < 0.05, log_2_ FoldChange ≤ −1, and number of sgRNA reads per gene > 2) (Fig. 1C; Table S3). Then, we took the intersection of the MAGeCK‐VISPR and edgeR results, which yielded a subset of 81 highly confident erlotinib‐sensitizing genes (Fig. 1D; Table S4). These 81 genes constitute the main candidate genes whose knockout led to decreased cell viability after erlotinib treatment, thus serving as the foundation for identifying new targets to overcome erlotinib resistance.

### 3.2 Functional characterization of 81 candidate genes reveals cell cycle and ubiquitination as key regulatory processes

In order to understand the overall biological roles of the putative erlotinib‐sensitizing genes, we performed network and functional analyses for these 81 genes. An expanded molecular network of protein–protein interactions was obtained using the StringApp of Cytoscape [20], and a network topology analysis identified a highly connected subnetwork that consisted of genes associated with cell cycle processes and regulation of protein ubiquitination (Fig. 1E). Notably, two genes (ANAPC5 and CDK1) out of the 81 candidate genes were related to both biological processes, suggesting interplay of the two processes. Similarly, we also performed a gene set enrichment analysis for these 81 candidate genes using Gene Ontology (GO) terms for biological processes in MSigDB (http://software.broadinstitute.org/gsea/index.jsp), which again identified mitotic cell cycle and regulation of protein ubiquitination as enriched processes (Fig. S2).

**Fig. 2:**
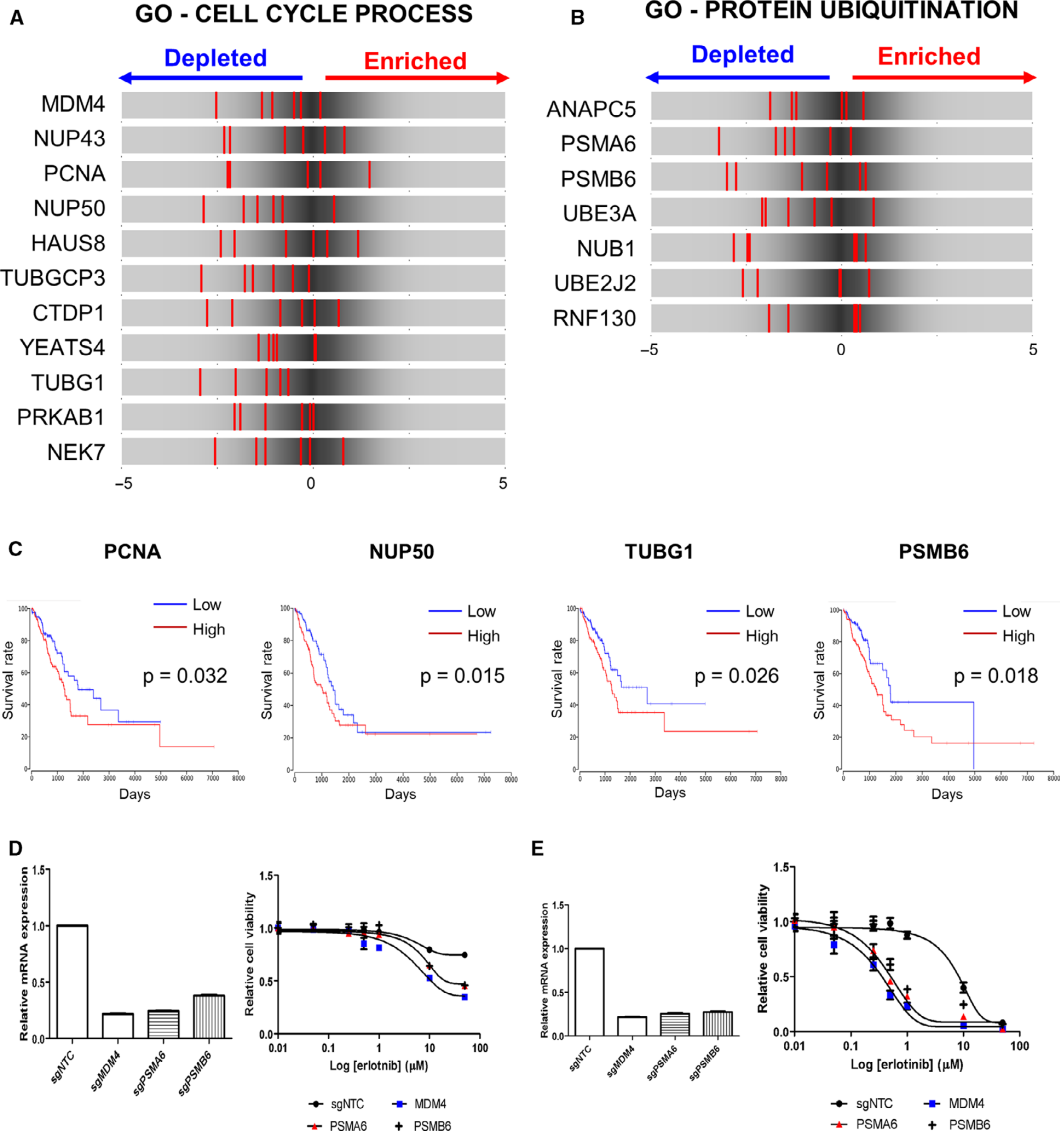
Loss‐of‐function screening identifies targets that were depleted in erlotinib resistance cells. (A, B) The rank distribution diagram of sgRNAs targeting genes associated with cell cycle processes (A) and protein ubiquitination (B). Ranks of sgRNAs (shown as the background) were obtained from the log_2_ ratio of the normalized sgRNA counts between the control and erlotinib treatment samples at day 14. The ranks of 6 sgRNAs targeting the gene of interest are shown with red vertical lines. (C) Survival analysis according to gene expression of candidate genes, using the TCGA‐LUAD patient cohort (*n* = 492). Patient groups of high and low expression indicate the top (*n* = 123) and bottom (*n* = 123) quartiles, respectively, according to gene expression level of interest. *P*‐values were obtained from the log‐rank test. (D) The validation experiments of single CRISPR/Cas9 treatment about candidate gene in NCI‐H820 cells. The mRNA expression levels of target gene were detected by real‐time PCR and the sensitivity of erlotinib was determined with dose–response curves. The erlotinib IC_50_ decreased for each candidate sgRNA‐transduced cell line compared to control sgRNA transduce cell (sgNTC). (E) The knockout of candidate genes had more effected in other lung cancer cell line NCI‐H1975. The IC_50_ of erlotinib to each sgRNA‐transduced cells was highly decreased in NCI‐H1975 cells.

We found that 11 (13.1%, *P = *0.027) and 7 (8.6%, *P = *0.046) out of 81 genes were assigned to ‘cell cycle process’ and ‘protein ubiquitination’ GO terms, respectively (Fig. 2A,B). Since our GeCKO library consists of 6 sgRNAs per gene, we examined the rank distribution of sgRNAs for genes in these two processes to check for knockout efficiency and consistency of sgRNAs. The rank position plot showed that the majority of sgRNAs for these genes were located on the depletion side (Fig. 2A,B). Thus, our results strongly suggest that knockout of these genes, involved in cell cycle or protein ubiquitination, can transform the erlotinib‐resistant tumor cells to become sensitive to erlotinib.

To study the clinical significance of these genes in the cell cycle process and protein ubiquitination pathways, we further performed Kaplan–Meier survival analyses based on the gene expression level of 492 lung adenocarcinoma patients from the TCGA database [24]. We compared the overall survival of patients in the top *vs*. bottom quartile of the target gene’s expression. Among 11 cell cycle process genes and 7 protein ubiquitination genes, we found that the prognosis was significantly better (*P* < 0.05) in patients with lower expression of three cell cycle‐related genes, PCNA, NUP50, and TUBG1, and one protein ubiquitination gene, PSMB6 (Fig. 2C). Thus, expression levels of these genes could serve as potential prognostic markers in NSCLC.

Additionally, we performed validation experiments to determine whether loss of the candidate genes contributes to erlotinib sensitivity in the lung cancer cells. NCI‐H820 and NCI‐H1975 cells were infected with lentivirus carrying sgRNAs targeting candidate gene and treated with various doses of erlotinib. Cell viability was determined after 3 days to evaluate the drug resistance. sgRNAs targeting MDM4, PSMA6, and PSMB6 led to a decrease in mRNA expression and increased sensibility to erlotinib (Fig. 2D,E). The IC50 of erlotinib to each sgRNA‐transduced cell increased 13‐fold (MDM4), 6.9‐fold (PSMA6), and 3.7‐fold (PSMB6), respectively, compared to control cells in NCI‐H820 (Fig. 2D). Dose–response curves for NCI‐H1975 cells, transduced with individual targeting lentiCRISPR, show that loss of MDM4, PSMA6, or PSMB6 conferred the most significant sensitivity to erlotinib (Fig. 2E). We also compared to the erlotinib IC50 for cells that targeted ANAPC5 or CDK1 or control sgRNA. The erlotinib IC50 was decreased for each target gene in the respective NCI‐H1975 knockout cells (Fig. S3).

### 3.3 Pharmacological inhibition of targets in cell cycle or ubiquitination pathways exhibited synergistic effect

**Fig. 3:**
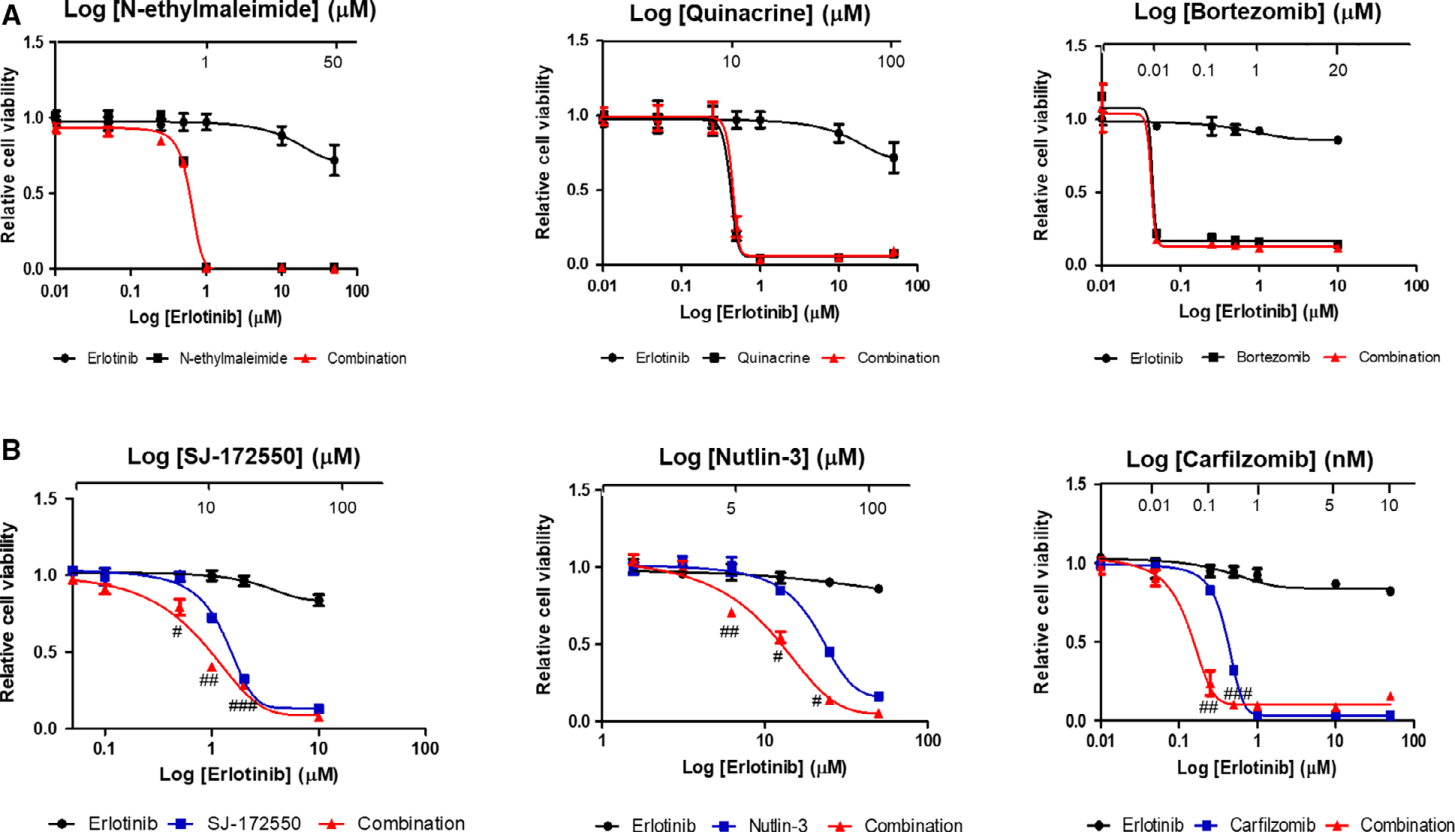
Cytotoxicity of erlotinib and three chemical inhibitors. (A) N‐ethylmaleimide, quinacrine, and bortezomib effectively inhibited the growth of erlotinib‐resistant NCI‐H820, with or without erlotinib. (B) SJ‐172550, nutlin‐3, and carfilzomib show a synergistic effect with erlotinib. Erlotinib‐resistant NCI‐H820 cells were used in viability assays. WST assays were used to examine the cell viability. Data are represented as mean values ± standard error of mean (SEM) based on triplicate experiments, and the regression lines were calculated by dose–response inhibition models in prism 5.0. Combination index (CI) values were calculated at applied concentrations, and the pound signs represent synergistic effect of the two drugs (#, CI < 0.9; ##, CI < 0.6; ###, CI < 0.3).

The effect of gene knockout by sgRNAs can also be achieved by chemical inhibitors with sufficient specificity. To investigate the efficacy of combination therapies to overcome erlotinib resistance, we searched for chemical inhibitors targeting the 81 candidate genes from our CRISPR screen. Through extensive literature and database surveys, thirteen chemical inhibitors were found to target these candidate genes (Table S5). Unfortunately, there were no commercial inhibitors for the two highly confident targets, ANAPC5 and CDK1, which appear to have functions in both cell cycle and protein ubiquitination processes. We tested the drug efficacy and the synergistic effect with erlotinib for each of the thirteen chemical inhibitors on the NCI‐H820 lung cancer cell line in an *in vitro* setting (Fig. S4A). Six of the inhibitors were shown to affect cell viability in a dose‐dependent manner. Three chemicals (N‐ethylmaleimide for ACAD8, quinacrine for MB21D1, and bortezomib for PSMA6 and PSMB6) were effective as a monotherapy with no significant additional benefits in combination treatments with erlotinib (Fig. 3A). Three other drugs (SJ‐172550 for MDM4, nutlin‐3 for MDM2/MDM4, and carfilzomib for PSMA6 and PSMB6) showed improved IC50 values when used as a monotherapy but increased efficacy when used in combination with erlotinib on NCI‐H820 lung cancer cell lines (Fig. 3B). Validation experiments in another erlotinib‐resistant lung cancer cell line, NCI‐H1975, confirmed the synergistic effect of these latter three inhibitors (i.e., SJ‐172550, nutlin‐3, and carfilzomib) with erlotinib (Fig. S4B). Thus, our data demonstrated that targeting certain genes in the ‘Cell cycle process’ (MDM4/MDM2 with SJ‐172550 and nutlin‐3) and ‘Protein ubiquitination’ GO term (PSMA6 and PSMB6 with carfilzomib) were effective in enhancing responsiveness to erlotinib. Interestingly, we found that the combination of nutlin‐3 or carfilzomib and osimertinib (3rd‐generation inhibitors of EGFR T790M) were highly effective in EGFR T790M lung cancer cell line NCI‐H1975 (Fig. S5).

### 3.4 Activation of the p53 pathway with nutlin‐3 confers erlotinib sensitivity by inducing cell cycle arrest and apoptosis

We identified MDM4 as one of the candidate genes involved in cell cycle (Fig. 2) through our sgRNA screen. The major function of MDM4 is to suppress p53 activities by MDM4‐p53 interaction. Interestingly, the MDM4 inhibitor, SJ‐172550, and the MDM2 inhibitor, nutlin‐3, showed a synergistic effect with erlotinib in erlotinib‐resistant lung cancer cell lines, NCI‐H820 (Fig. 3B) and NCI‐H1975 (Fig. S4B).

**Fig. 4:**
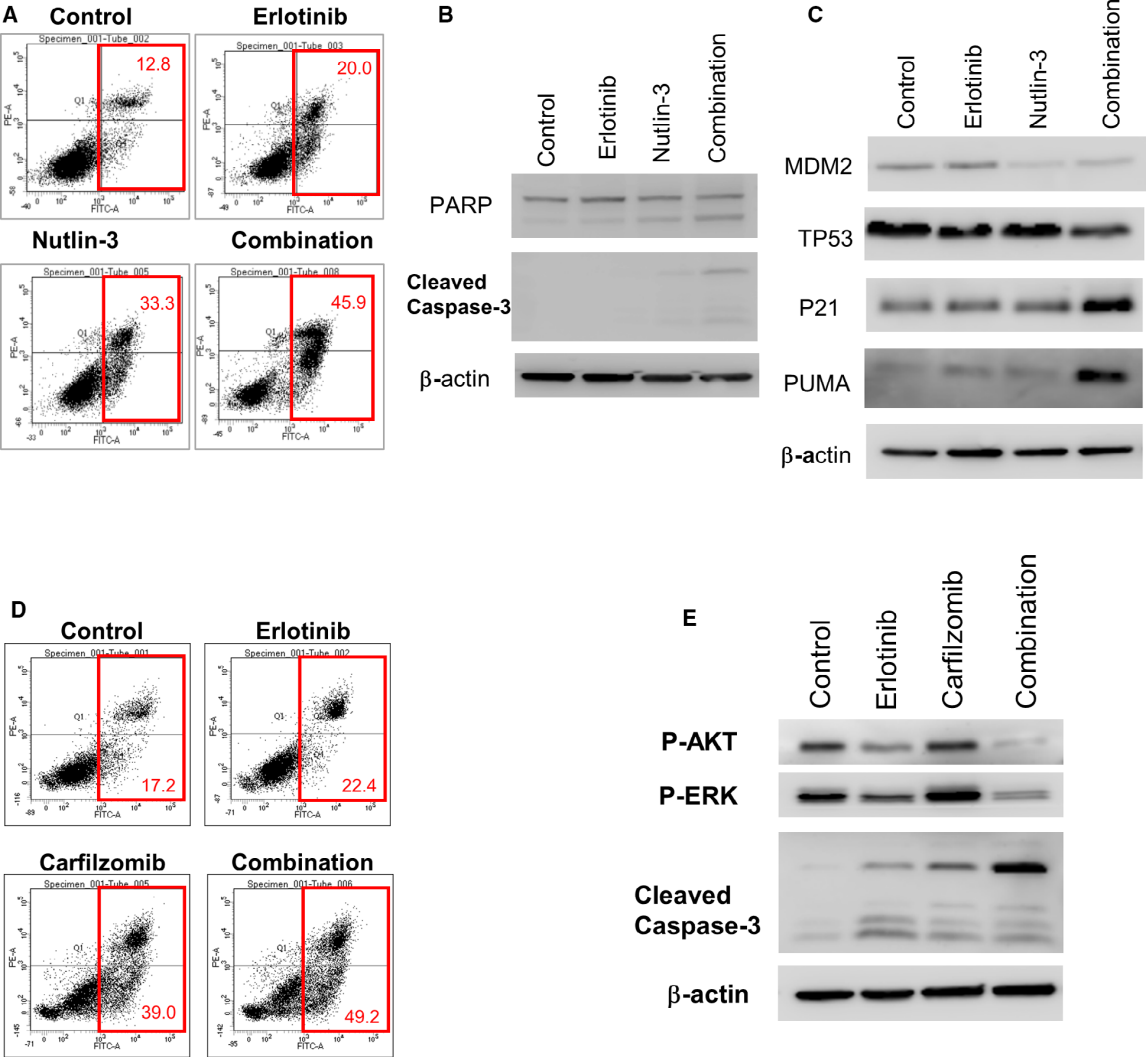
Synergistic effect of erlotinib and target gene’s inhibitor in the erlotinib‐resistant lung cancer cell line, NCI‐H820. (A) The combination of erlotinib and nutlin‐3 increases cell death, as demonstrated by flow cytometry analysis and (B) cleavage of poly (ADP‐ribose) polymerase (PARP) and caspase 3 expression in NCI‐H820 cells. (C) Activation of the p53 pathway with nutlin‐3 augmented erlotinib response by inducing apoptosis via PUMA expression. (D) Combination of erlotinib and carfilzomib increases cell death demonstrated by flow cytometry analysis and (E) cleavage of caspase‐3, phospo‐AKT, and phosphor‐ERK protein expression. All experiments were performed in duplicate, each being repeated at least two times.

We focused on nutlin‐3, because the apoptosis effect was more prominent in the combination of nutlin‐3 and erlotinib, compared to SJ‐172550 and erlotinib, based on propidium iodide staining (Figs 4A, S6A). The combination treatment of erlotinib and nutlin‐3 resulted in a significant increase of cleavage of the poly (ADP‐ribose) polymerase (PARP) and caspase 3 (Fig. 4B), which are characteristics of apoptotic cell death. In addition, the combination of erlotinib and nutlin‐3 increased the expression of the p21 protein (Fig. 4C), which is a strong inducer of cell cycle arrest and increased PUMA (p53 upregulated modulator of apoptosis) expression (Fig. 4C), which is a pro‐apoptotic protein which induces apoptosis by inhibiting anti‐apoptotic Bcl‐2 family proteins [25]. The expression of PUMA is already known to be regulated by p53 and is involved in p53‐dependent and p53‐independent apoptosis induced by a variety of signals, ultimately leading to cell death [26]. Taken together, activation of the p53 pathway with nutlin‐3 appears to augment the response of erlotinib by inducing both cell cycle arrest and apoptosis via p21 and PUMA induction, respectively (Fig. 4C). Additionally, we confirmed that the molecular phenotype in MDM4 knockout cells mimics the phenotype in nutlin‐3‐treated cells in erlotinib‐treated condition (Fig. S6B). A pro‐apoptotic protein PUMA expression was increased in erlotinib‐treated MDM4 knockout cell.

Carfilzomib and erlotinib combination treatments also showed synergistic effects in apoptotic cell death (Fig. 4D). The increase of cell death (17.2% to 49.2%; 2.86‐fold) was higher in combination therapy compared to monotherapies with erlotinib (17.2% to 22.4%; 1.30‐fold) or carfilzomib alone (17.2% to 39.0%; 2.27‐fold). Additionally, the cleavage of caspase 3 in NCI‐H820 cells was increased dramatically during combination treatments with both erlotinib and carfilzomib compared to erlotinib or carfilzomib treatments alone (Fig. 4E). Interestingly, phosphorylation of AKT and ERK was significantly decreased for the combination treatment on NCI‐H820 cells (Fig. 4E). Phosphorylated AKT and ERK inhibit apoptosis and induce cell survival through phosphorylation of various proteins such as Bad, GSK3β, caspase 9, and FoxO transcription factors [27]. These results suggest that the combination treatment with erlotinib and carfilzomib also synergistically induces cell death in erlotinib‐resistant cells.

### 3.5 Synergistic effects of erlotinib‐sensitizing chemicals were validated in in vivo patient‐derived xenografts (PDXs) mouse models

**Fig. 5:**
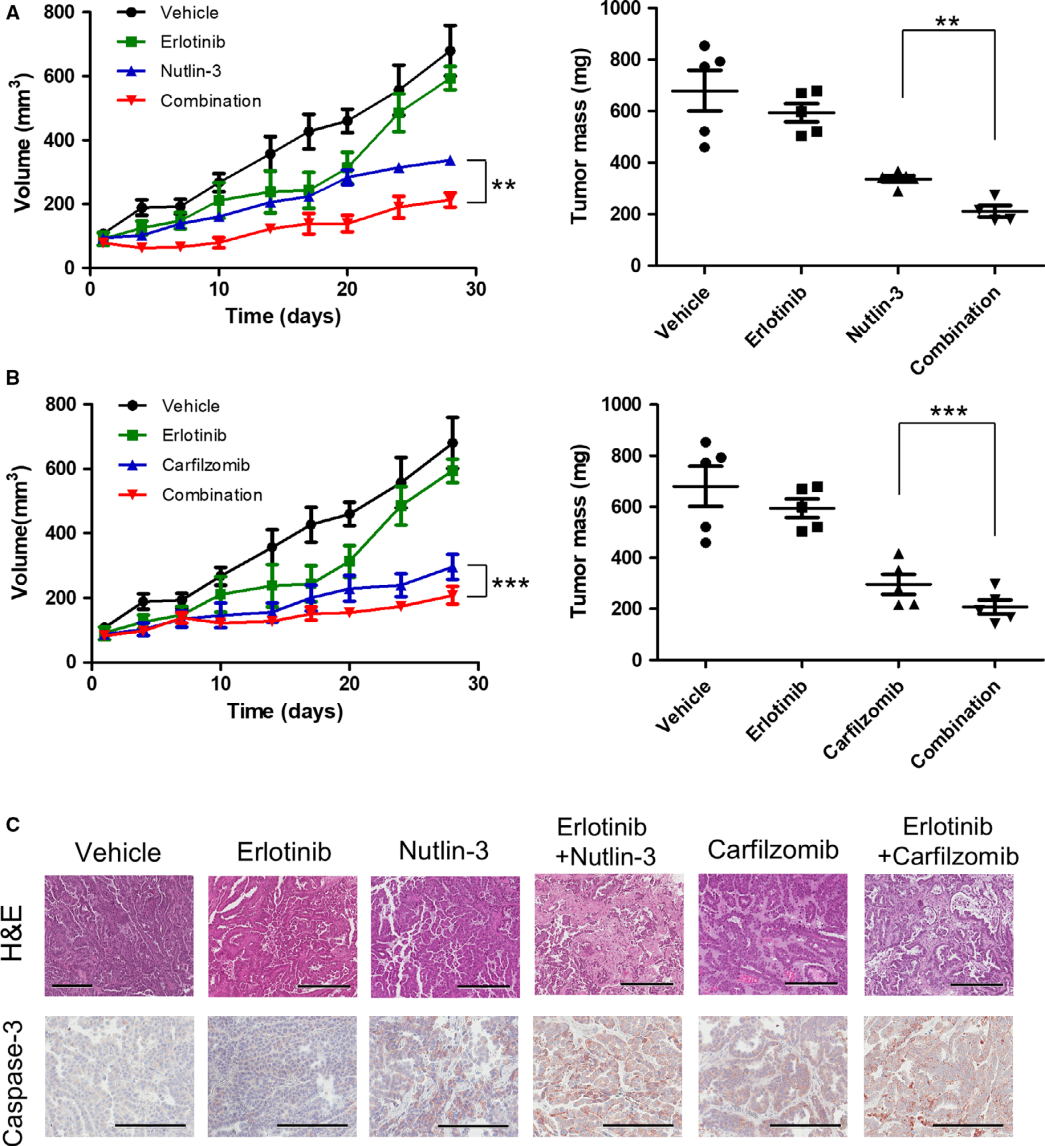
*In vivo* efficacy of erlotinib‐based combination treatment with nutlin‐3 or carfilzomib in EGFR T790M mutated patient‐derived xenografts. Tumor volumes of each group’s mice (*N* = 5) are shown on the left and their tumor mass after 28 days of treatment are shown on the right. (A) The synergistic effect of erlotinib and nutlin‐3 in lung adenocarcinoma mouse models with the EGFR T790M mutation (LG1049). A significant effect upon tumor growth was observed with combinations of erlotinib and nutlin‐3 (2‐way ANOVA: **, *P = *0.0069) when compared with nutlin‐3 only. Similarly, when the mass of the tumors at endpoint were compared, the combinations of erlotinib and nutlin‐3 (unpaired *t*‐test: **, *P = *0.0014) showed significant improvement over nutlin‐3. (B) The synergistic effect of erlotinib and carfilzomib in lung adenocarcinoma mouse models with the EGFR T790M mutation (LG1049). A significant effect upon tumor growth was observed with combinations of erlotinib and carfilzomib (2‐way ANOVA: ***, *P* < 0.0001) when compared with carfilzomib only. Similarly, when the mass of the tumors at endpoint were compared, the combinations of erlotinib and carfilzomib (unpaired *t*‐test: ***, *P* < 0.0001) showed significant improvements over carfilzomib alone. Graphs represent mean ± SD of the experiments. (C) Representative H&E‐stained section and IHC against the apoptosis marker cleaved caspase‐3 of representative tumors from each of the treatment groups; scale bar, 300 μm. The xenografts that have been treated with a combination of erlotinib and nutlin‐3 or erlotinib and carfilzomib showed increased cleaved caspase 3 expression.

To further study these three putative erlotinib‐sensitizing chemicals (SJ‐172550, nutlin‐3, and carfilzomib), we performed efficacy testing with a human lung cancer PDX model having an EGFR T790M mutation (i.e., comprised of erlotinib‐resistant cells). The LG1049 PDX was implanted in immunodeficient NSG mice and subsequently treated with erlotinib, SJ‐172550, nutlin‐3, or carfilzomib as well as with treatments combining erlotinib with SJ‐172550, nutlin‐3, or carfilzomib. Nutlin‐3 and carfilzomib both showed significant cell death when used alone or in combination with erlotinib (Fig. 5A,B) but treatment with SJ‐172550 alone did not show a significant tumor inhibitory growth effect in these *in vivo* experiments (Fig. S7). The combination of erlotinib with nutlin‐3 or carfilzomib increased apoptosis signals by cleaved caspase 3 staining (Fig. 5C). These results are in good concordance with the previous *in vitro* cell experiment data for both monotherapy and combination treatments. Taken together, combinatorial inhibition of nutlin‐3 and erlotinib or carfilzomib and erlotinib was effective in tumor growth inhibition of an erlotinib‐resistant lung cancer PDX.

## 4 Discussion

A pooled CRISPR‐Cas sgRNA library screen can provide new opportunities to systematically identify new targets for drug resistance. We conducted a large‐scale loss‐of‐function screen in a lung cancer cell line, using this CRISPR‐Cas9 system, to find new genes associated with erlotinib responsiveness and to understand mechanisms underlying resistance to this drug.

In this study, we identified two major pathways that contribute to erlotinib resistance. The first target pathway was cell cycle processes. It is known that p53 is eventually involved in cancer development by stimulating or inhibiting cell cycle regulators, so it is very important to understand the cell cycle regulatory processes leading to cancer development.

The mutations of *TP53* gene were a negative prognostic factor for overall survival (OS) of advanced non‐small‐cell lung cancer (NSCLC) patients, and, in EGFR‐mutated patients, additional mutations of *TP53* gene were associated with shorter OS [28]. In addition, in *EGFR*‐mutated NSCLC, comutation of *TP53*, especially missense mutations, exhibited the trend toward shorter progression‐free survival on EGFR tyrosine kinase inhibitors (TKIs) [29], suggesting that functional p53 is associated with the responsiveness of EGFR‐TKIs. In molecular levels, functional p53 was reported to enhance another EGFR inhibitor, gefitinib‐induced growth arrest through the up‐regulation of Fas in lung cancer cells [30]. These previous reports suggest that cell cycle regulation by functional p53 plays considerable roles in the determination of the responsiveness to erlotinib. Nutlin‐3 is a small molecule inhibitor of the MDM2/p53 interaction, which leads to nongenotoxic p53 stabilization as well as activation of cell cycle arrest and apoptosis pathways [31]. Since the discovery of the nutlins in 2004, several compounds have been developed and shown to target the p53‐MDM2/4 axis by inhibiting MDM2 and/or MDM4 [32]. Nutlin‐3 has a synergistic cytotoxic effect when used in combination with TNF‐related apoptosis‐inducing ligand (TRAIL), bortezomib, and cisplatin to induce p53‐dependent tumor cell apoptosis in NSCLC [31, 33]. Nutlin‐3 preferentially sensitizes wild‐type p53‐expressing cancer cells such as the A549 lung cancer cell line [34], but our study shows that it also appears to work in mutant p53‐expressing cancer cells (TP53 T284P in the NCI‐H820 cell line; TP53 R273H in the NCI‐H1975 cell line; and TP53 P72R in the LG1049 PDX model). In addition, nutlin‐3 synergizes with erlotinib in both an erlotinib‐resistant lung cancer cell lines NCI‐H820, NCI‐H1975, and *in vivo* in an erlotinib‐resistant lung cancer PDX model (Fig. 3B and Fig. 5A). The MDM4 inhibitor, SJ‐172550, also showed synergistic cell death when used in combination with erlotinib in an *in vitro* assay but this synergistic effect was not reproduced *in vivo* in mouse drug tests (Fig. S5). Identifying inhibitors specific to ubiquitin‐proteasome pathways is usually of great interest for cancer treatment because aberrations in the components of this pathway are commonly observed in many cancers and uncontrolled growth of cancer cells can result either from stabilization of oncoproteins (e.g., c‐jun) or increased degradation of tumor suppressor proteins (e.g., p53).

Protein ubiquitination and proteasomal pathway are an evolutionary conserved process for protein degradation, and crucial for biological processes, including cell survival and differentiation [35]. Several tumor‐promoting or suppressing pathways, including NF‐κB, HIF‐1α, and p53 pathways, are regulated through protein ubiquitination processes [35]. The EGFR protein itself was regulated via ubiquitinated, and deubiquitinating enzyme, ubiquitin‐specific protease 22 (USP22) was associated with EGFR‐TKI resistance by enhancing EGFR stability [36]. Inhibition of the ubiquitin‐proteasome pathway exerts antitumor effect by regulating several key pathways, including inhibition of NF‐κB pathway via increase of I‐κB, inhibition of cell cycle via increase of p53 and cyclins, and increase of apoptosis via increased of p53‐mediated Bax protein [37], and these pathways are associated with the responsiveness to EGFR‐TKIs [30, 38]. Carfilzomib is a second‐generation anticancer drug acting as a selective proteasome inhibitor. It irreversibly binds and inhibits the chymotrypsin‐like activity of the 20S proteasome, an enzyme that degrades unwanted cellular proteins. Inhibition of proteasome‐mediated protein ubiquitination results in a buildup of polyubiquitinated proteins, which is thought to cause cell cycle arrest, apoptosis, and inhibition of tumor growth [39, 40]. Carfilzomib was approved by the U.S. FDA in 2012 for multiple myeloma patients who have received at least two prior therapies, including treatment with bortezomib and an immunomodulatory therapy (such as lenalidomide), and have demonstrated disease progression on or within 60 days of completion of the last therapy [39]. Furthermore, the U.S. FDA has approved carfilzomib, in combination with other anticancer drugs, to treat patients with relapsed multiple myeloma. However, its efficacy has not yet been tested in clinical trials of lung cancer. Here, we show that a combination treatment using erlotinib and carfilzomib could efficiently induce apoptosis of erlotinib resistance lung cancer cells.

Over the years, various trials have been attempted to identify new drugs to overcome erlotinib resistance. For example, a second‐generation EGFR‐TKI, an irreversible inhibitor that contains a Michael‐acceptor group and covalently attaches to the EGFR ATP binding site, may act as a pan‐HER inhibitor that also inhibits EGFR, HER2, and HER4 [41]. Additionally, a third‐generation EGFR inhibitor that specifically responds to T790M EGFR, such as osimertinib, has been recently approved by the U.S. FDA [42]. Moreover, mutant EGFR, including T790M, is more sensitive to an inhibitor of the heat‐shock protein 90 (HSP90) than normal EGFR carrying cells [43]. Thus, combination of an HSP90 inhibitor and TKI could be another way to effectively overcome T790M resistance. Finally, animal studies using T790M transgenic mice have shown that the combination of EGFR blocking antibodies cetuximab and BIBW2992 can also be effective in overcoming T790M resistance [44]. Although third‐generation inhibitors are available in the clinic, lst‐generation EGFR‐TKIs, such as erlotinib and gefitinib, are still widely used in first‐line treatment of NSCLC patients with EGFR mutations. In addition, candidate chemicals from our screening showed synergistic effect with 3rd‐generation inhibitor, osimertinib in some EGFR‐mutated NSCLC cells (Fig. S5), suggesting that our screens are probably applicable for enhancing the effect of 3rd‐generation inhibitors.

## 5 Conclusions

Erlotinib is highly effective in lung cancer patients with EGFR mutations. However, despite initial favorable responses, many patients develop erlotinib resistance. Here, we applied a CRISPR/cas9 library screen to identify five genes (MDM4, PSMA6, PSMB6, ANAPC5, and CDK1) that are associated with erlotinib responsiveness and have provided evidence that inhibitors for two of these genes (nutlin‐3 for MDM4 or carfilzomib for PSMA6 and PSMB6) can be used in combination treatments with erlotinib to potentially minimize the development of erlotinib resistance. We hypothesize that targeting cell cycle processes with nutlin‐3, or protein ubiquitination processes with carfilzomib, might be promising new clinical strategies to overcome resistance to EGFR inhibitor drugs in NSCLC.

## Conflict of interest

The authors declare no conflict of interest.

## Author contributions

JL, AC, S‐YC, SL, and CL involved in study concept and design; JL, AC, S‐YC, YJ, DN, and AL contributed to acquisition of data. JL, AC, S‐YC, and YJ analyzed and interpreted the data. JL, AC, S‐YC, GJ, JYK, JK, SL, and CL involved in critical revision of the manuscript; SL and CL supervised the study. All authors edited and approved the manuscript.

### Peer Review

The peer review history for this article is available at https://publons.com/publon/10.1002/1878‐0261.12853.

## Supporting information


**Fig. S1.** CRISPR/cas9 screening results of all conditions.
**Fig. S2.** Gene Ontology (GO) analysis using MSigDB.
**Fig. S3.** Validation experiments on selected two genes, ANAPC5 and CDK1.
**Fig. S4.** The test of drug efficacy and the synergistic effect with erlotinib for each of the thirteen chemical inhibitors on the NCI‐H820 lung cancer cell line in an *in vitro* setting.
**Fig. S5.** Synergistic effect of 3^rd^ generation TKIs inhibitor osimertinib and nutlin‐3 or carfilzomib in the erlotinib resistant lung cancer cell line NCI‐H1975.
**Fig. S6.** Synergistic effect of erlotinib and SJ‐172550 in the erlotinib resistant lung cancer cell line, NCI‐H820.
**Fig. S7.** *In vivo* efficacy of erlotinib‐based combination treatment with SJ‐172550 in EGFR T790M mutated patient‐derived xenografts.

**Table S1.** The sgRNA sequence for target gene validation.
**Table S2.** The beta score from MAGeCK‐VISPR program for highly variable genes used in k‐means clustering.
**Table S3.** List of 1945 genes with differentially expressed sgRNAs at day 14.
**Table S4.** The MAGeck‐VISPR and EdgR analysis showed a subset of 81 genes.
**Table S5.** The druggable candidate genes and related inhibitors.

## Data Availability

The Gene Expression Omnibus accession numbers for sgRNA sequencing data are GSE142669.

## References

[1] Jemal A , Bray F , Center MM , Ferlay J , Ward E & Forman D (2011) Global cancer statistics. Ca‐Cancer J Clin 61, 69–90. 21296855 10.3322/caac.20107

[2] Wang ZX (2016) Transactivation of epidermal growth factor receptor by G protein‐coupled receptors: recent progress, challenges and future research. Int J Mol Sci 17, 95. 10.3390/ijms17010095 PMC4730337 26771606

[3] Hynes NE & Lane HA (2005) ERBB receptors and cancer: the complexity of targeted inhibitors. Nat Rev Cancer 5, 341–354. 15864276 10.1038/nrc1609

[4] Thomas A , Rajan A & Giaccone G (2012) Tyrosine kinase inhibitors in lung cancer. Hematol Oncol Clin 26, 589–605. 10.1016/j.hoc.2012.02.001 PMC3334853 22520981

[5] Wang Y , Schmid‐Bindert G & Zhou C (2012) Erlotinib in the treatment of advanced non‐small cell lung cancer: an update for clinicians. Ther Adv Med Oncol 4, 19–29. 22229045 10.1177/1758834011427927 PMC3244201

[6] Bartholomew C , Eastlake L , Dunn P & Yiannakis D (2017) EGFR targeted therapy in lung cancer; an evolving story. Respir Med Case Rep 20, 137–140. 28217439 10.1016/j.rmcr.2017.01.016 PMC5302182

[7] Shepherd FA , Pereira JR , Ciuleanu T , Tan EH , Hirsh V , Thongprasert S , Campos D , Maoleekoonpiroj S , Smylie M , Martins R *et al* (2005) Erlotinib in previously treated non‐small‐cell lung cancer. New Engl J Med 353, 123–132. 16014882 10.1056/NEJMoa050753

[8] Lee JG & Wu R (2012) Combination erlotinib‐cisplatin and Atg3‐mediated autophagy in erlotinib resistant lung cancer. PLoS One 7, e48532. 23119048 10.1371/journal.pone.0048532 PMC3485310

[9] Morgillo F , Della Corte CM , Fasano M & Ciardiello F (2016) Mechanisms of resistance to EGFR‐targeted drugs: lung cancer. ESMO Open 1, e000060. 10.1136/esmoopen-2016-000060 PMC5070275 27843613

[10] Engelman JA , Zejnullahu K , Gale CM , Lifshits E , Gonzales AJ , Shimamura T , Zhao F , Vincent PW , Naumov GN , Bradner JE *et al* (2007) PF00299804, an irreversible pan‐ERBB inhibitor, is effective in lung cancer models with EGFR and ERBB2 mutations that are resistant to gefitinib. Cancer Res 67, 11924–11932. 18089823 10.1158/0008-5472.CAN-07-1885

[11] Takezawa K , Pirazzoli V , Arcila ME , Nebhan CA , Song X , de Stanchina E , Ohashi K , Janjigian YY , Spitzler PJ , Melnick MA *et al* (2012) HER2 amplification: a potential mechanism of acquired resistance to EGFR inhibition in EGFR‐mutant lung cancers that lack the second‐site EGFRT790M mutation. Cancer Discov 2, 922–933. 22956644 10.1158/2159-8290.CD-12-0108 PMC3473100

[12] Barrangou R & Doudna JA (2016) Applications of CRISPR technologies in research and beyond. Nat Biotechnol 34, 933–941. 27606440 10.1038/nbt.3659

[13] Jinek M , Chylinski K , Fonfara I , Hauer M , Doudna JA & Charpentier E (2012) A Programmable dual‐RNA‐guided DNA endonuclease in adaptive bacterial immunity. Science 337, 816–821. 22745249 10.1126/science.1225829 PMC6286148

[14] Hsu PD , Lander ES & Zhang F (2014) Development and applications of CRISPR‐Cas9 for genome engineering. Cell 157, 1262–1278. 24906146 10.1016/j.cell.2014.05.010 PMC4343198

[15] Mullenders J & Bernards R (2009) Loss‐of‐function genetic screens as a tool to improve the diagnosis and treatment of cancer. Oncogene 28, 4409–4420. 19767776 10.1038/onc.2009.295

[16] de la Fuente‐Nunez C & Lu TK (2017) CRISPR‐Cas9 technology: applications in genome engineering, development of sequence‐specific antimicrobials, and future prospects. Integr Biol (Camb) 9, 109–122. 28045163 10.1039/c6ib00140h

[17] Shalem O , Sanjana NE , Hartenian E , Shi X , Scott DA , Mikkelson T , Heckl D , Ebert BL , Root DE , Doench JG *et al* (2014) Genome‐scale CRISPR‐Cas9 knockout screening in human cells. Science 343, 84–87. 24336571 10.1126/science.1247005 PMC4089965

[18] Cho SY , Han JY , Na D , Kang W , Lee A , Kim J , Lee J , Min S , Kang J , Chae J *et al* (2017) A novel combination treatment targeting BCL‐XL and MCL1 for KRAS/BRAF‐mutated and BCL2L1‐amplified colorectal cancers. Mol Cancer Ther 16, 2178–2190. 28611106 10.1158/1535-7163.MCT-16-0735

[19] Sanjana NE , Shalem O & Zhang F (2014) Improved vectors and genome‐wide libraries for CRISPR screening. Nat Methods 11, 783–784. 25075903 10.1038/nmeth.3047 PMC4486245

[20] Doncheva NT , Morris JH , Gorodkin J & Jensen LJ (2019) Cytoscape StringApp: network analysis and visualization of proteomics data. J Proteome Res 18, 623–632. 30450911 10.1021/acs.jproteome.8b00702 PMC6800166

[21] Liberzon A , Subramanian A , Pinchback R , Thorvaldsdottir H , Tamayo P & Mesirov JP (2011) Molecular signatures database (MSigDB) 3.0. Bioinformatics 27, 1739–1740. 21546393 10.1093/bioinformatics/btr260 PMC3106198

[22] Pauli C , Hopkins BD , Prandi D , Shaw R , Fedrizzi T , Sboner A , Sailer V , Augello M , Puca L , Rosati R *et al* (2017) Personalized in vitro and in vivo cancer models to guide precision medicine. Cancer Discov 7, 462–547. 28331002 10.1158/2159-8290.CD-16-1154 PMC5413423

[23] Li W , Koster J , Xu H , Chen CH , Xiao T , Liu JS , Brown M & Liu XS (2015) Quality control, modeling, and visualization of CRISPR screens with MAGeCK‐VISPR. Genome Biol 16, 281. 26673418 10.1186/s13059-015-0843-6 PMC4699372

[24] Anaya J (2016) OncoLnc: linking TCGA survival data to mRNAs, miRNAs, and lncRNAs. Peerj Comput Sci 2, e67.

[25] Nakano K & Vousden KH (2001) PUMA, a novel proapoptotic gene, is induced by p53. Mol Cell 7, 683–694. 11463392 10.1016/s1097-2765(01)00214-3

[26] Han J , Flemington C , Houghton AB , Gu Z , Zambetti GP , Lutz RJ , Zhu L & Chittenden T (2001) Expression of bbc3, a pro‐apoptotic BH3‐only gene, is regulated by diverse cell death and survival signals. Proc Natl Acad Sci U S A 98, 11318–11323. 11572983 10.1073/pnas.201208798 PMC58727

[27] Jung HJ & Suh Y (2014) Regulation of IGF ‐1 signaling by microRNAs. Front Genet 5, 472. 25628647 10.3389/fgene.2014.00472 PMC4292735

[28] Jiao XD , Qin BD , You P , Cai J & Zang YS (2018) The prognostic value of TP53 and its correlation with EGFR mutation in advanced non‐small cell lung cancer, an analysis based on cBioPortal data base. Lung Cancer 123, 70–75. 30089598 10.1016/j.lungcan.2018.07.003

[29] Labbe C , Cabanero M , Korpanty GJ , Tomasini P , Doherty MK , Mascaux C , Jao K , Pitcher B , Wang R , Pintilie M *et al* (2017) Prognostic and predictive effects of TP53 co‐mutation in patients with EGFR‐mutated non‐small cell lung cancer (NSCLC). Lung Cancer 111, 23–29. 28838393 10.1016/j.lungcan.2017.06.014

[30] Rho JK , Choi YJ , Ryoo BY , Na II , Yang SH , Kim CH & Lee JC (2007) p53 enhances gefitinib‐induced growth inhibition and apoptosis by regulation of Fas in non‐small cell lung cancer. Cancer Res 67, 1163–1169. 17283151 10.1158/0008-5472.CAN-06-2037

[31] Secchiero P , Bosco R , Celeghini C & Zauli G (2011) Recent advances in the therapeutic perspectives of Nutlin‐3. Curr Pharm Des 17, 569–577. 21391907 10.2174/138161211795222586

[32] Tisato V , Voltan R , Gonelli A , Secchiero P & Zauli G (2017) MDM2/X inhibitors under clinical evaluation: perspectives for the management of hematological malignancies and pediatric cancer. J Hematol Oncol 10, 133. 28673313 10.1186/s13045-017-0500-5 PMC5496368

[33] Deben C , Wouters A , Op de Beeck K , van Den Bossche J , Jacobs J , Zwaenepoel K , Peeters M , Van Meerbeeck J , Lardon F , Rolfo C *et al* (2015) The MDM2‐inhibitor Nutlin‐3 synergizes with cisplatin to induce p53 dependent tumor cell apoptosis in non‐small cell lung cancer. Oncotarget 6, 22666–22679. 26125230 10.18632/oncotarget.4433 PMC4673190

[34] Meijer A , Kruyt FA , van der Zee AG , Hollema H , Le P , ten Hoor KA , Groothuis GM , Quax WJ , de Vries EG & de Jong S (2013) Nutlin‐3 preferentially sensitises wild‐type p53‐expressing cancer cells to DR5‐selective TRAIL over rhTRAIL. Br J Cancer 109, 2685–2695. 24136147 10.1038/bjc.2013.636 PMC3833221

[35] Popovic D , Vucic D & Dikic I (2014) Ubiquitination in disease pathogenesis and treatment. Nat Med 20, 1242–1253. 25375928 10.1038/nm.3739

[36] Zhang H , Han B , Lu H , Zhao Y , Chen X , Meng Q , Cao M , Cai L & Hu J (2018) USP22 promotes resistance to EGFR‐TKIs by preventing ubiquitination‐mediated EGFR degradation in EGFR‐mutant lung adenocarcinoma. Cancer Lett 433, 186–198. 29981430 10.1016/j.canlet.2018.07.002

[37] Rajkumar SV , Richardson PG , Hideshima T & Anderson KC (2005) Proteasome inhibition as a novel therapeutic target in human cancer. J Clin Oncol 23, 630–663. 15659509 10.1200/JCO.2005.11.030

[38] Stanam A , Love‐Homan L , Joseph TS , Espinosa‐Cotton M & Simons AL (2015) Upregulated interleukin‐6 expression contributes to erlotinib resistance in head and neck squamous cell carcinoma. Mol Oncol 9, 1371–1383. 25888065 10.1016/j.molonc.2015.03.008 PMC4523436

[39] Herndon TM , Deisseroth A , Kaminskas E , Kane RC , Koti KM , Rothmann MD , Habtemariam B , Bullock J , Bray JD , Hawes J *et al* (2013) U.S. Food and Drug Administration approval: carfilzomib for the treatment of multiple myeloma. Clin Cancer Res 19, 4559–4563. 23775332 10.1158/1078-0432.CCR-13-0755

[40] Khan ML & Stewart AK (2011) Carfilzomib: a novel second‐generation proteasome inhibitor. Future Oncol 7, 607–612. 21568676 10.2217/fon.11.42 PMC3931449

[41] Kwak EL , Sordella R , Bell DW , Godin‐Heymann N , Okimoto RA , Brannigan BW , Harris PL , Driscoll DR , Fidias P , Lynch TJ *et al* (2005) Irreversible inhibitors of the EGF receptor may circumvent acquired resistance to gefitinib. Proc Natl Acad Sci U S A 102, 7665–7670. 15897464 10.1073/pnas.0502860102 PMC1129023

[42] Gao X , Le X & Costa DB (2016) The safety and efficacy of osimertinib for the treatment of EGFR T790M mutation positive non‐small‐cell lung cancer. Expert Rev Anticancer Ther 16, 383–390. 26943236 10.1586/14737140.2016.1162103 PMC4940973

[43] Shimamura T , Li D , Ji H , Haringsma HJ , Liniker E , Borgman CL , Lowell AM , Minami Y , McNamara K , Perera SA *et al* (2008) Hsp90 inhibition suppresses mutant EGFR‐T790M signaling and overcomes kinase inhibitor resistance. Cancer Res 68, 5827–5838. 18632637 10.1158/0008-5472.CAN-07-5428 PMC3272303

[44] Regales L , Gong Y , Shen R , de Stanchina E , Vivanco I , Goel A , Koutcher JA , Spassova M , Ouerfelli O , Mellinghoff IK *et al* (2009) Dual targeting of EGFR can overcome a major drug resistance mutation in mouse models of EGFR mutant lung cancer. J Clin Invest 119, 3000–3010. 19759520 10.1172/JCI38746 PMC2752070

