# Supplementary material for: Genome‐scale CRISPR screening identifies cell cycle and protein ubiquitination processes as druggable targets for erlotinib‐resistant lung cancer
Source: Mol Oncol. 2020 Nov 28;15(2):487–502. doi: 10.1002/1878-0261.12853 (PMC7858278; doi:10.1002/1878-0261.12853)
Supplement: Supplementary file 1 — Fig. S1. CRISPR/cas9 screening results of all conditions. Fig. S2. Gene Ontology (GO) analysis using MSigDB. Fig. S3. Validation experiments on selected two genes, ANAPC5 and CDK1. Fig. S4. The test of drug efficacy and the synergistic effect with erlotinib for each of the thirteen chemical inhibitors on the NCI‐H820 lung cancer cell line in an in vitro setting. Fig. S5. Synergistic effect of 3rd generation TKIs inhibitor osimertinib and nutlin‐3 or carfilzomib in the erlotinib resistant lung cancer cell line NCI‐H1975. Fig. S6. Synergistic effect of erlotinib and SJ‐172550 in the erlotinib resistant lung cancer cell line, NCI‐H820. Fig. S7. In vivo efficacy of erlotinib‐based combination treatment with SJ‐172550 in EGFR T790M mutated patient‐derived xenografts. [file MOL2-15-487-s001.zip › mol212853-sup-0001-Legends.docx]

MOLONC-20-0299

**Supplementary Figure Legends**

**Fig. S1. CRISPR/cas9 screening results of all conditions.** (A) Box plot and (B) correlation plot of the sgRNA for all conditions. The log2-transformed normalized counts were used. The replicates (duplicate in all conditions) show excellent reproducibility in both plots. Positive and negative selections are evident in control and erlotinib-treated samples in 14 days.

**Fig. S2. Gene Ontology (GO) analysis using MSigDB.** Statistical enrichment in GO terms on biological processes was investigated for 81 candidate genes using MSigDB analysis tool (http://software.broadinstitute.org/gsea/index.jsp) (FDR < 0.05). Representative terms are cell cycle and protein ubiquitination processes as in the Cytoscape StringApp analysis.

**Fig. S3. validation experiments on selected two genes, ANAPC5 and CDK1.** we checked whether loss of function of these genes confer erlotinib sensitivity in the lung cancer cells. The erlotinib sensitivity increased in ANAPC5 or CDK1 knockout NCI-H820 cells (P < 0.05).

**Fig. S4. The test of drug efficacy and the synergistic effect with erlotinib for each of the thirteen chemical inhibitors on the NCI-H820 lung cancer cell line in an *in vitro* setting.** (A) We searched for chemical inhibitors targeting the 81 candidate genes from our CRISPR screen and tested the efficacy and the synergistic effect with erlotinib and eleven drugs. The check mark (✓) means the drug had a synergistic effect with erlotinib. (B) We confirmed that the synergistic effect of three inhibitors (i.e., SJ-172550, nutlin-3 and carfilzomib) with erlotinib in another erlotinib-resistant lung cancer cell line NCI-H1975. Combination index (CI) values were calculated at applied concentrations and the pound signs represent synergistic effect of the two drugs (#, CI < 0.9; ##, CI < 0.6; ###, CI < 0.3).

**Fig. S5. Synergistic effect of 3^rd^ generation TKIs inhibitor osimertinib and nutlin-3 or carfilzomib in the erlotinib resistant lung cancer cell line NCI-H1975.** The combination of nutlin-3 or carfilzomib with osimertinib were highly effective in EGFR T790M lung cancer cell line NCI-H1975. Combination index (CI) values were calculated at applied concentrations and the pound signs represent synergistic effect of the two drugs (###, CI < 0.3).

**Fig. S6. Synergistic effect of erlotinib and SJ-172550 in the erlotinib resistant lung cancer cell line, NCI-H820.** (A) The combination of erlotinib and SJ-172550 increased cell death, as demonstrated by flow cytometry analysis and (B) a pro-apoptotic protein PUMA expression in NCI-H820 cells.

**Fig. S7*. In vivo* efficacy of erlotinib-based combination treatment with SJ-172550 in EGFR T790M mutated patient-derived xenografts.** Tumor volumes of these mice are shown on the left and their tumor mass after 28 days of treatment are shown on the right. (A) The statistical synergistic effect of tumor growth in erlotinib and SJ-172550 was not observed in lung adenocarcinoma mouse models with the EGFR T790M mutation (LG1049) (2-way ANOVA: *P =* 1.000) when compared with SJ-172550 only. Similarly, when the mass of the tumors at endpoint were compared, the combinations of erlotinib and SJ-172550 (Unpaired *t* test: *P =* 0.5967) not showed significant improvement over SJ-172550.
