# Supplementary figures and images for: Genome‐scale CRISPR screening identifies cell cycle and protein ubiquitination processes as druggable targets for erlotinib‐resistant lung cancer
Source: Mol Oncol. 2020 Nov 28;15(2):487–502. doi: 10.1002/1878-0261.12853 (PMC7858278; doi:10.1002/1878-0261.12853)

A

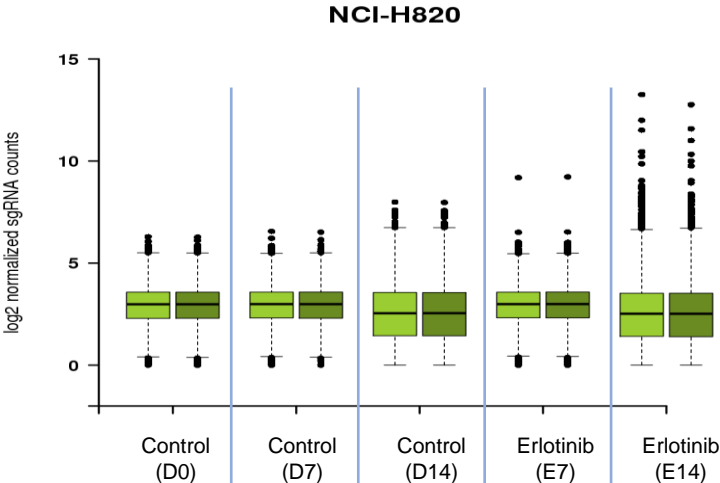

B

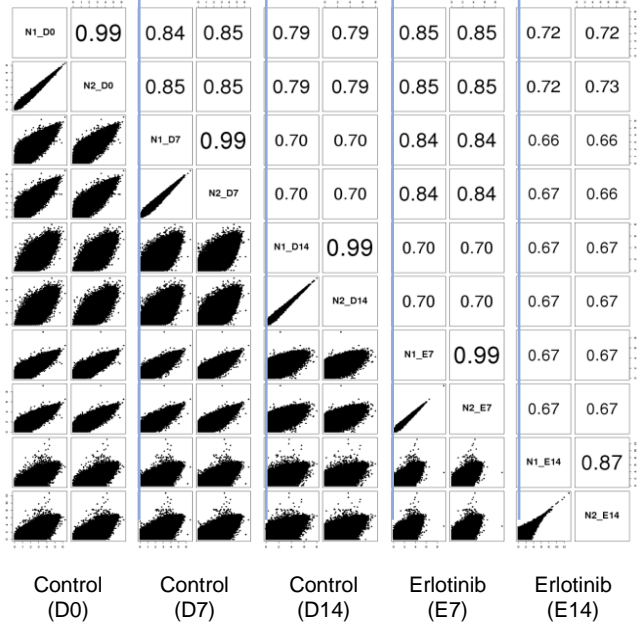

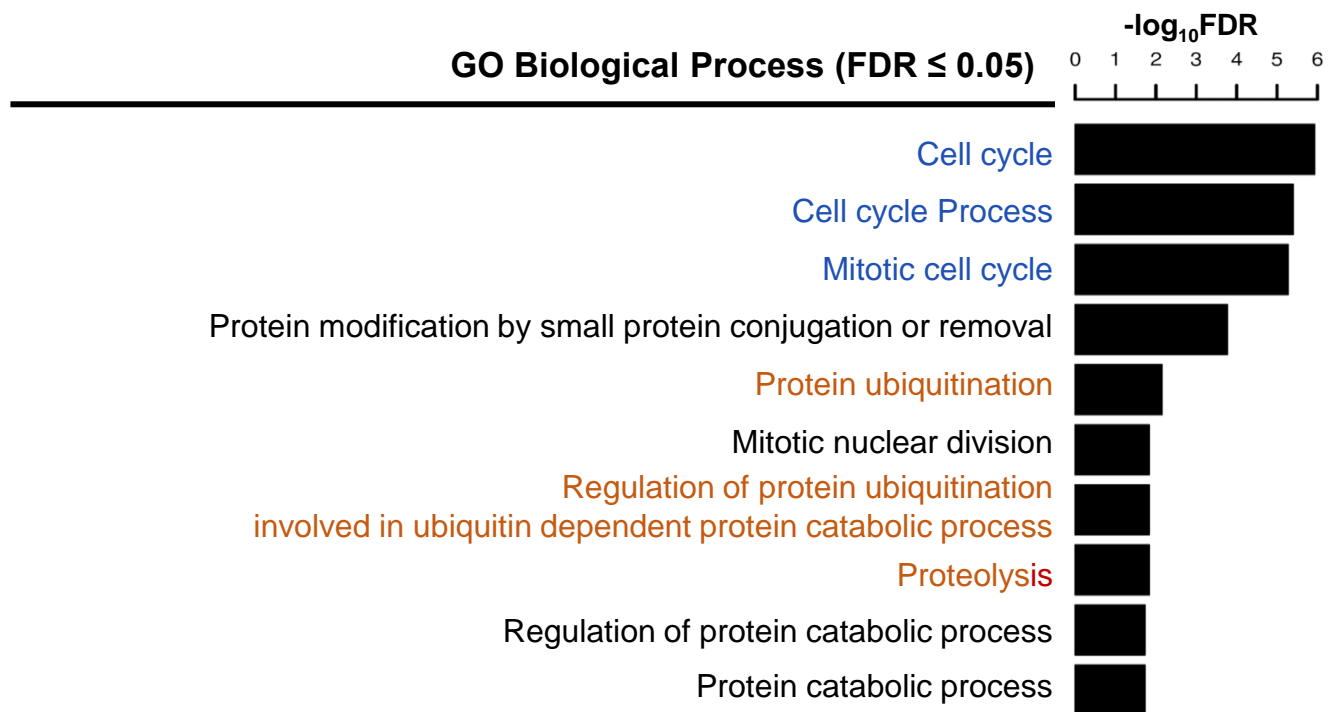

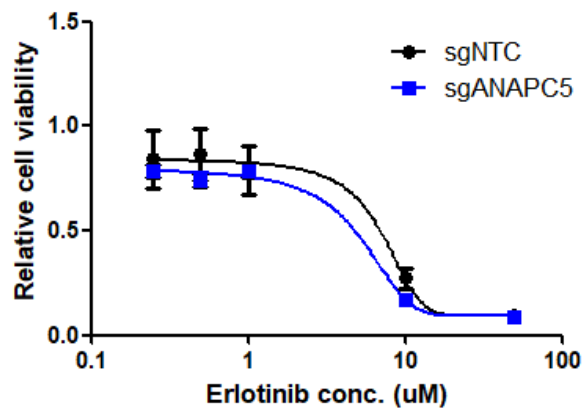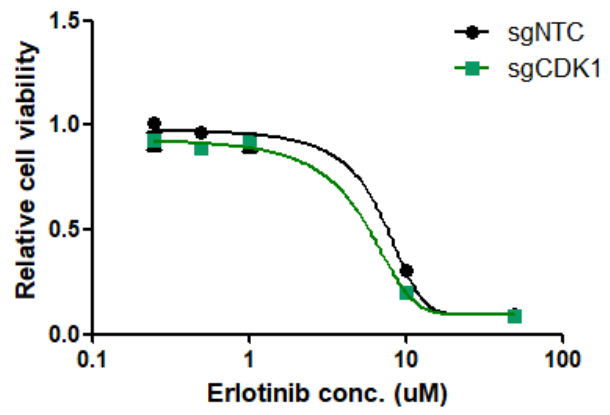

A

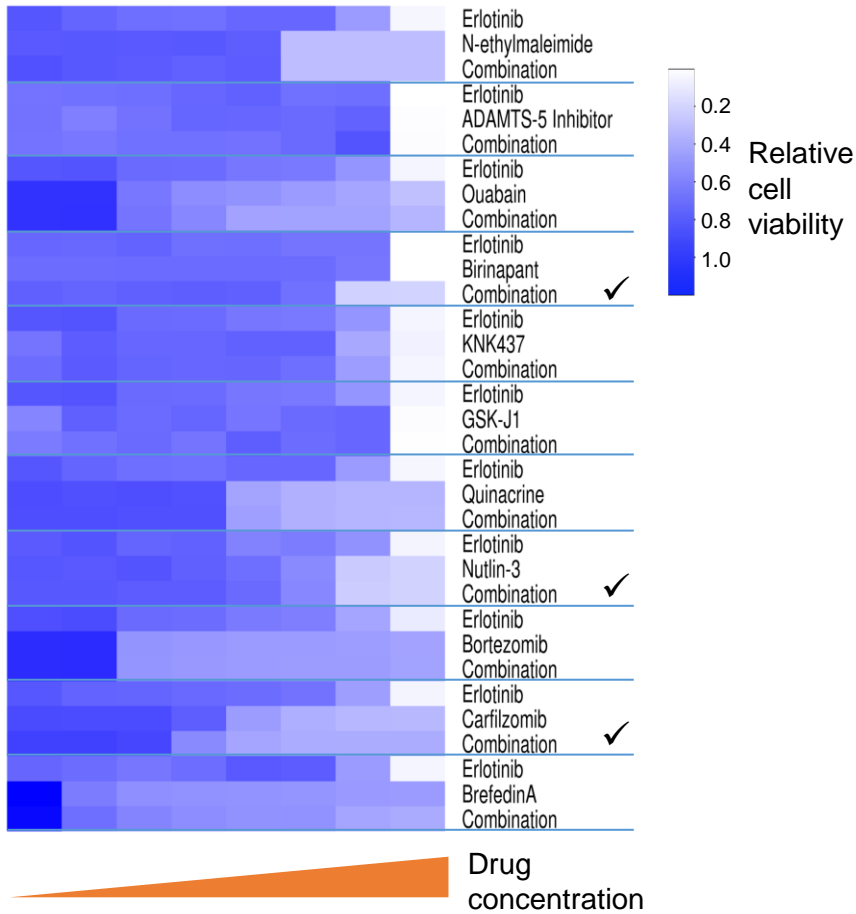

B

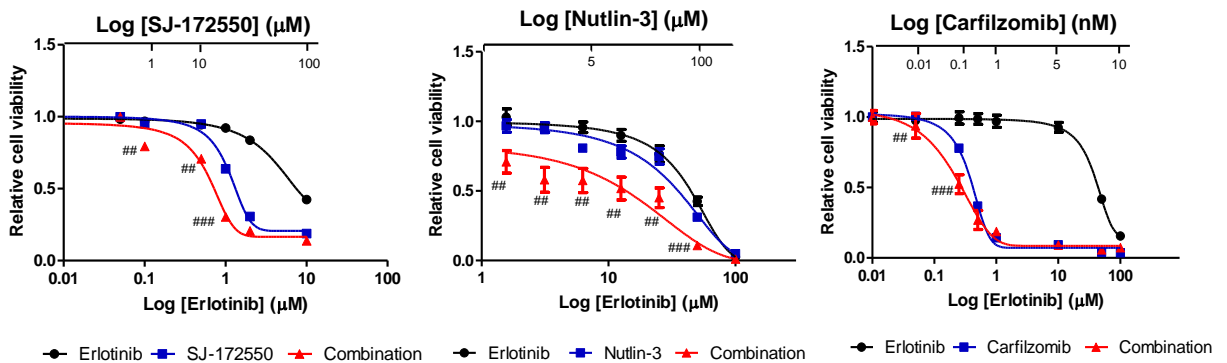

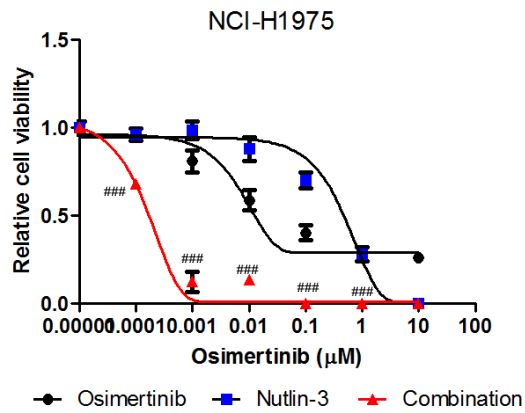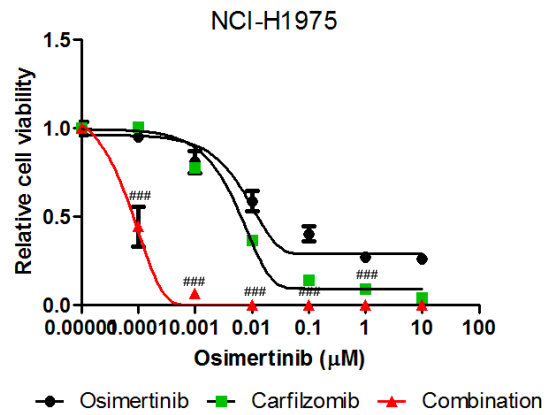

**A**

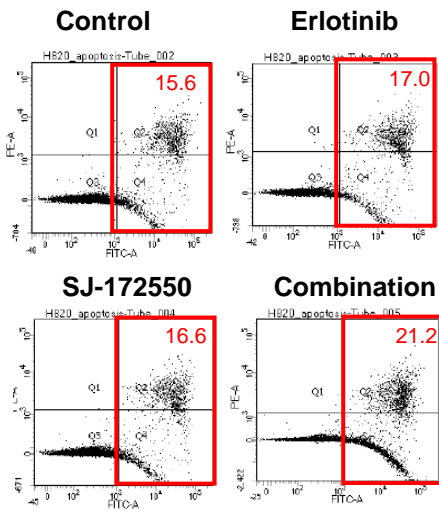

**B**

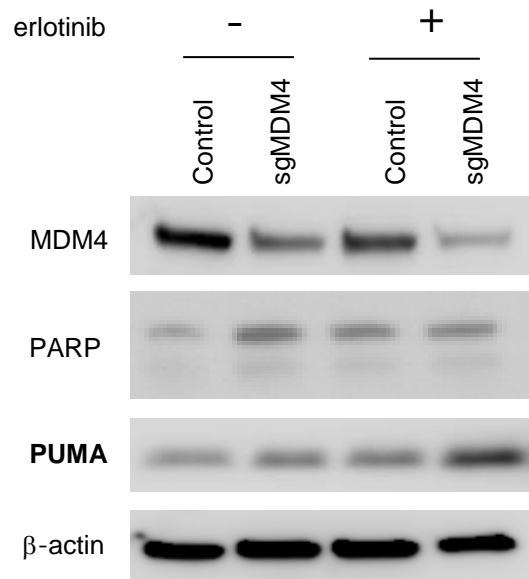

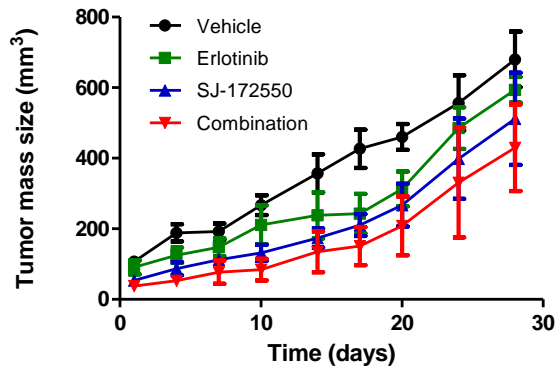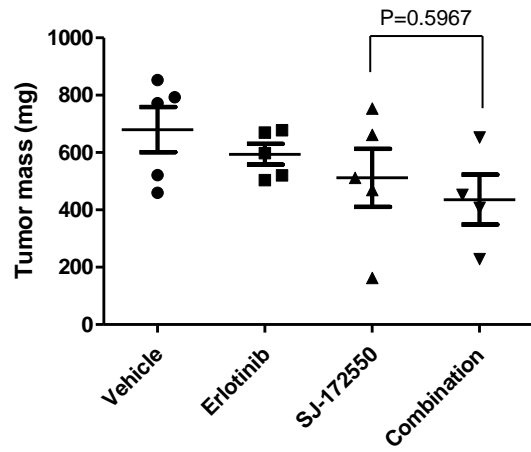

Supplement: Supplementary file 1 — Fig. S1. CRISPR/cas9 screening results of all conditions. Fig. S2. Gene Ontology (GO) analysis using MSigDB. Fig. S3. Validation experiments on selected two genes, ANAPC5 and CDK1. Fig. S4. The test of drug efficacy and the synergistic effect with erlotinib for each of the thirteen chemical inhibitors on the NCI‐H820 lung cancer cell line in an in vitro setting. Fig. S5. Synergistic effect of 3rd generation TKIs inhibitor osimertinib and nutlin‐3 or carfilzomib in the erlotinib resistant lung cancer cell line NCI‐H1975. Fig. S6. Synergistic effect of erlotinib and SJ‐172550 in the erlotinib resistant lung cancer cell line, NCI‐H820. Fig. S7. In vivo efficacy of erlotinib‐based combination treatment with SJ‐172550 in EGFR T790M mutated patient‐derived xenografts. [file MOL2-15-487-s001.zip › mol212853-sup-0001-FigS1-S7.pdf]
